# Early introduction of peanut reduces peanut allergy across risk groups in pooled and causal inference analyses

**DOI:** 10.1111/all.15597

**Published:** 2022-12-11

**Authors:** Kirsty Logan, Henry T. Bahnson, Alyssa Ylescupidez, Kirsten Beyer, Johanna Bellach, Dianne E. Campbell, Joanna Craven, George Du Toit, E. N. Clare Mills, Michael R. Perkin, Graham Roberts, Ronald van Ree, Gideon Lack

**Affiliations:** ^1^ Department of Women and Children's Health, School of Life Course and Population Sciences King's College London London UK; ^2^ Immune Tolerance Network Benaroya Research Institute Seattle Washington USA; ^3^ Charité Universitätsmedizin Berlin Berlin Germany; ^4^ Children's Hospital at Westmead Westmead New South Wales Australia; ^5^ School of Biological Sciences, Division of Infection, Immunity and Respiratory Medicine, Manchester Academic Health Science Centre, Manchester Institute of Biotechnology University of Manchester Manchester UK; ^6^ The Population Health Research Institute St George's University of London London UK; ^7^ University of Southampton and Southampton NIHR Biomedical Research Centre Southampton UK; ^8^ Departments of Experimental Immunology and of Otorhinolaryngology Amsterdam University Medical Centers Amsterdam Netherlands

**Keywords:** causal inference analysis, early introduction, peanut allergy prevention

## Abstract

**Background:**

The Learning Early About Peanut allergy (LEAP) study has shown the effectiveness of early peanut introduction in prevention of peanut allergy (PA). In the Enquiring About Tolerance (EAT) study, a statistically significant reduction in PA was present only in per‐protocol (PP) analyses, which can be subject to bias.

**Objective:**

The aim of this study was to combine individual‐level data from the LEAP and EAT trials and provide robust evidence on the bias‐corrected, causal effect of early peanut introduction.

**Method:**

As part of the European Union‐funded iFAAM project, this pooled analysis of individual pediatric patient data combines and compares effectiveness and efficacy estimates of oral tolerance induction among different risk strata and analysis methods.

**Results:**

An intention‐to‐treat (ITT) analysis of pooled data showed a 75% reduction in PA (*p* < .0001) among children randomized to consume peanut from early infancy. A protective effect was present across all eczema severity groups, irrespective of enrollment sensitization to peanut, and across different ethnicities. Earlier age of introduction was associated with improved effectiveness of the intervention. In the pooled PP analysis, peanut consumption reduced the risk of PA by 98% (*p* < .0001). A causal inference analysis confirmed the strong PP effect (89% average treatment effect relative risk reduction *p* < .0001). A multivariable causal inference analysis approach estimated a large (100%) reduction in PA in children without eczema (*p* = .004).

**Conclusion:**

We demonstrate a significant reduction in PA with early peanut introduction in a large group of pooled, randomized participants. This significant reduction was demonstrated across all risk subgroups, including children with no eczema. Furthermore, our results point to increased efficacy of the intervention with earlier age of introduction.

## Graphical abstract

An integrated meta‐analysis employing both LEAP (high risk) and EAT (normal risk) individual patient data shows that early introduction of peanut in infancy prevents the development of peanut allergy regardless of presence or severity of eczema, ethnicity and sensitization to peanut. Earlier introduction of peanut before 6 months of age is more effective than later introduction in the prevention of peanut allergy. Causal inference methods accurately estimated the efficacy of this strategy while addressing the problems of poor adherence in the intention‐to‐treat analyses and biases in the per‐protocol analyses.Abbreviations: EAT, enquiring about tolerance; ITT, intention to treat; LEAP, learning early about allergy; PP, per protocol

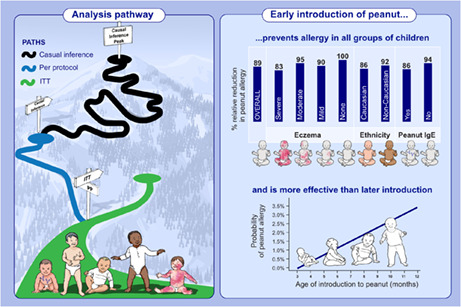

## 1 INTRODUCTION

Despite strong findings favoring early peanut introduction, there are gaps in our knowledge base, especially with respect to whether to target high risk or normal populations and age of intervention. It is therefore not surprising that specialist organizations provide different guidelines regarding peanut introduction. Most recently, the European Academy of Allergy and Clinical Immunology (EAACI) guidelines gave a conditional recommendation for the introduction of peanut to the whole infant population as the strength of the evidence was considered to be moderate.^1^ These recommendations were limited to countries with a high prevalence of peanut allergy. In order to increase our knowledge base for future guidelines, we conducted an integrated analysis of individual participant data from all available randomized controlled trials (RCT) of peanut introduction.

Until recently, UK and US guidelines recommended avoidance of peanut in infancy to prevent the development of peanut allergy.^2^, ^3^ Despite these guidelines, the prevalence of peanut allergy continued to increase and subsequently a growing body of evidence emerged favoring early introduction of peanut rather than early avoidance.

An observational study comparing Israeli and UK children found that the early introduction and regular consumption of peanut in infancy was strongly protective against developing peanut allergy.^4^ A series of early introduction RCTs have subsequently taken place. There have been two independently powered and randomized UK‐based cohorts investigating the early introduction of peanut.^5^, ^6^ A recent systematic review and meta‐analysis of these two early introduction RCTs were undertaken, and the pooled risk ratio for peanut introduction was 0.29 (0.11–0.74).^7^ The review concluded that the current body of evidence supported the early introduction of peanut for the prevention of peanut allergy but was unable to investigate subgroups, such as infants with varying eczema severity, ethnicity and sensitization.^7^

The Learning Early About Peanut allergy (LEAP) study recruited infants at high risk of developing peanut allergy aged 4–10 months and showed an 81% relative reduction in peanut allergy prevalence between the peanut consumption and avoidance groups.^5^ The Enquiring About Tolerance (EAT) study enrolled children at 3 months of age and randomized them to either consume six allergenic foods including peanut (Early Introduction Group: EIG) or to avoid allergenic food consumption until 6 months (Standard Introduction Group: SIG). A 51% reduction in peanut allergy prevalence was observed but did not reach statistical significance in the intention‐to‐treat (ITT) analysis.^6^ Adherence to peanut consumption in the LEAP study was 92%. In comparison, peanut adherence was significantly lower in the EAT study (61% of 549 peanut adherence evaluable participants and 48% of all 652 EIG participants). The reasons for poor adherence in the EAT study have been extensively investigated previously where increasing maternal age, non‐Caucasian ethnicity and lower maternal quality of life were found to be important factors influencing adherence. In comparison with LEAP study participants, the EAT participants introduced five other allergenic foods alongside peanut making the intervention harder to follow.^6^, ^8^

Despite the LEAP study demonstrating that earlier age of introduction of peanut reduced peanut allergy in high‐risk participants and subsequent changes to the American infant feeding guidelines,^9^ there remains uncertainty across clinical and policy spheres regarding the robustness of the results and their generalizability to lower risk populations. The EAT study set out to address some of those concerns by enrolling a lower risk cohort but ultimately did not demonstrate that the intervention had as strong of an effect in the ITT population. In particular, low protocol compliance in the EAT study led to complications in the interpretation of results.^6^

When adherence to an intervention is lacking, patients and investigators have been shown to prefer bias‐corrected estimates of an intervention's effectiveness across risk subgroups using valid per‐protocol (PP) effect estimates.^10^ Since ITT analyses estimate the effect of treatment assignment, not the actual treatment received, they can produce misleading causal estimates of an intervention when adherence is reduced. Therefore, effective interventions, diluted by non‐adherence, can appear to be ineffective using ITT analyses. For these reasons and others, alternative analysis approaches have been proposed to adjust for post‐randomization imbalances of treatment adherence.^11^ In addition to ITT and PP analyses of the pooled data from both cohorts, we have implemented causal inference analysis approaches to estimate the effect of early introduction of peanut among the different risk strata and adherence populations and to adjust PP analyses for biases often present due to post‐randomization imbalances of the intervention received rather than assigned. For example, a small number of participants in the intervention group had peanut allergy at baseline or developed peanut allergy while receiving the intervention and were subsequently instructed per the protocol not to begin or to discontinue consumption of peanut, respectively. In the PP analysis of the original study reports, these participants were considered non‐per‐protocol and thus were removed from the analysis. However, under a causal inference framework, these participants are included as protocol adherent participants and analyzed as receiving the intervention.^11^

Based on evidence from the LEAP study, and supported by results of the EAT study, the National Institute of Allergy and Infectious Diseases (NIAID) and a number of other specialist national societies issued guidelines recommending the early introduction of peanut for peanut allergy prevention. The evidence cited to support the NIAID recommendations in different eczema subgroups is based on those LEAP infants recruited with egg allergy (who had varying levels of eczema) and also from the EAT study participants, most of whom had no or mild to moderate eczema at enrollment.^9^

The pooled analysis of data from the LEAP and EAT cohorts presented here provides a unique opportunity to look at the effect of the early introduction of peanut across a variety of risk strata (presence of eczema and its severity, enrolment sensitization status to peanut and ethnicity), while implementing regression adjustment methodologies to estimate the causal effect of oral tolerance induction when adhered to.

## 2 METHODS

Study‐specific methods for the LEAP and EAT studies are published in full elsewhere and cohort demographics are summarized in Table S1.^12^, ^13^

The European Union‐funded iFAAM (Integrated Food Allergy and Allergen Management) project included a pooled analysis of individual‐level data from the RCTs of early allergenic food introduction. This pooled analysis allows estimates of oral tolerance induction among a number of different risk strata. Pooled estimates are adjusted for study‐specific and individual‐specific factors. Pooling the data across studies provides a robust and powerful estimate of the effect of oral tolerance induction. Analysis of the individual participant data enables estimates to be derived for the effect of early introduction of allergenic foods across the different ethnicities, baseline eczema severities and baseline IgE sensitization, a notable advantage in comparison with a meta‐analysis approach of summarized results.

Detailed methods and levels of adherence for the LEAP and EAT studies have been reported elsewhere. Data collected in each study included allergy, sensitization and anthropometric endpoints, as well as demographics and family history of atopy. The challenges of harmonizing data from studies that have differences in design and methodology were extensively discussed, and agreement was reached on what could feasibly be analyzed in one dataset and a statistical analysis plan outlined. Using the iFAAM‐funded Allerg‐e‐lab, each data point was annotated with descriptors, and individual datasets from each study were then recoded, renamed and relabelled using the agreed upon matched variables, thus creating individual study datasets containing identical names, formats, labels and coding values. The harmonized datasets were merged together as a final integrated dataset and analyzed using methods described below.

The outcomes evaluated were the point prevalence of peanut allergy by 3 years (EAT study primary endpoint) and by 5 years of age (LEAP study primary endpoint) and sensitization to peanut (measured as skin prick test [SPT] ≥1 mm or specific IgE ≥0.1 kU/L). Secondary outcome of baseline eczema, defined by objective Scoring Atopic Dermatitis (SCORAD) measurement, was also available from both studies.

The integrated dataset was validated to ensure published study results on primary and secondary outcomes from the individual trials could be replicated. All published data on peanut allergy and sensitization were replicated in the integrated dataset before combined analyses began.

### 2.1 Statistical analysis

The primary endpoint of this pooled analysis was peanut allergy prevalence—defined on the basis of a positive oral food challenge (OFC) or sensitization and symptom history where food challenge was not done. Ninety‐one per cent of peanut allergy diagnoses were made on the basis of OFC. The primary analysis methodology for the pooled endpoint was a logistic regression model. Univariate and multivariable adjustments to the risk of peanut allergy were made and shown as estimated probabilities of allergy, risk differences between the randomized groups and relative risk reductions calculated from the univariate and multivariable‐adjusted predicted probabilities from the logistic regression models. We pre‐specified a number of subgroups for comparison and the raw‐unadjusted proportions are shown according to the different pre‐specified subgroups. All tests were two‐sided at the alpha = 0.05 level of significance. Pearson's chi‐squared tests were also used to replicate individual study results. When one or more cells had expected counts less than 5, Fisher's exact test was used. Individual and combined study proportions were displayed as bar charts with frequencies, proportions and *p*‐values annotated. Relative risk reductions and risk differences with 95% confidence intervals were computed and displayed as forest plots among the analysis and study populations.

Causal inference methods were implemented to adjust PP analyses for biases often present due to post‐randomization imbalances of the intervention's uptake relative to assignment. The average treatment effect (ATE), which estimates the average treatment effect among the entire population, assumes each participant is able to receive the intervention. Doubly robust methods were used to estimate the ATE and 95% bootstrapped confidence intervals by combining inverse probability weighting and regression adjustment using the AIPW option and the bootstrap statement in SAS Proc Causaltrt.^14^, ^15^, ^16^ Estimates for the average treatment effect for the treated (ATT) and average treatment effect for the untreated (ATU) are reported in the Table S2 and Figure S3.

Causal inference PP analyses were performed using SAS software version 9.4 (Proc Causaltrt).^17^ The estimates were adjusted for ethnicity, baseline eczema severity and baseline peanut sensitization as covariates in each study separately and in the pooled study‐adjusted analysis to produce the ATE, ATT and ATU estimates. Complier average causal effect (CACE) was determined for each study and in a combined analysis adjusted for study using the R package ivpack version 1.2.^18^ The CACE analysis used an instrumental variable approach, with two‐staged least squares regression and active participation in the intervention as the predictor and randomized treatment assignment as the instrument. This instrumental variable approach produces marginal estimates of the treatment effect without making conditional adjustments for the covariates used in the SAS Proc Causaltrt approach.

## 3 RESULTS

Individual‐level participant data from the EAT and LEAP studies were combined to give a pooled estimate of peanut allergy prevalence in intervention and control groups based on data from 1943 children. Demographic characteristics, eczema severity and baseline IgE sensitization were balanced between control and intervention groups in the pooled data (Table S1).

### 3.1 Peanut allergy

**FIGURE 1:**
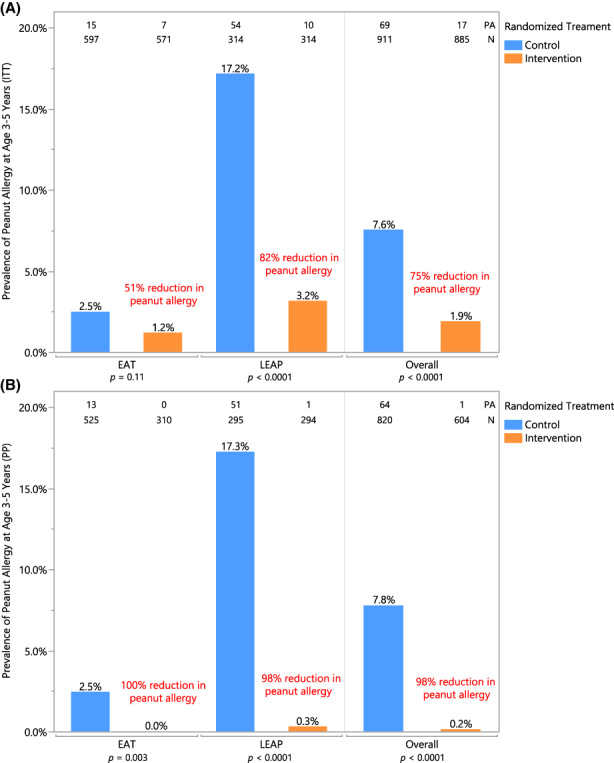
Peanut allergy prevalence at 3–5 years of age—(A) Intention‐to‐treat and (B) per‐protocol populations. Prevalence of peanut allergy in each randomized treatment group is shown within individual and combined studies in (A) ITT analysis and (B) PP analysis. Number of peanut allergy (PA) and total number of participants (*N*) are annotated at the top. Each bar is annotated with the prevalence of peanut allergy as determined by OFC at 3 years (EAT) or 5 years of age (LEAP); relative risk reduction in individual and combined studies is annotated in red. Pearson's chi‐squared was used to determine annotated *p*‐values; when expected values were less than 5, Fisher's two‐tailed exact test was used. Fisher's *p*‐value is reported for the EAT PP analysis.

Peanut allergy status could be evaluated in 1796 of 1943 children in the LEAP and EAT cohorts; 86 of whom (4.8%) were allergic to peanut at 3–5 years of age (Table S1). The ITT showed a 75% reduction in peanut allergy: 1.9% in the early introduction group versus 7.6% in the control group (*p* < .0001) (Figure 1A). Among those adherent to the early introduction or control protocols, the effect was strengthened with a 98% reduction in peanut allergy (*p* < .0001) (Figure 1B).

### 3.2 Eczema

Participants with eczema at baseline contributed disproportionately to the prevalence of peanut allergy: 0.9% (8/900) in those with no eczema versus 8.7% (78/895) in those with eczema. The effect of early introduction in these eczema subgroups is key to determining the potential impact of any early introduction strategy.

**FIGURE 2:**
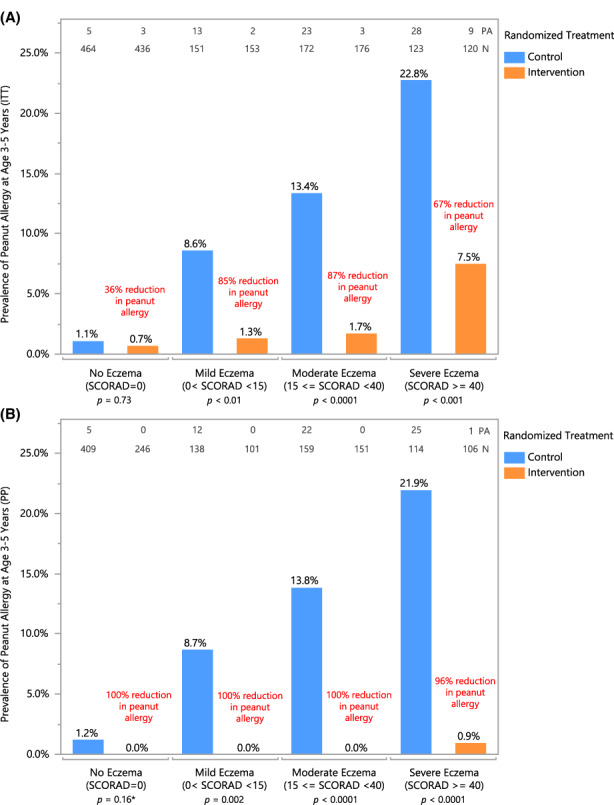
Peanut allergy prevalence by eczema severity—(A) intention‐to‐treat and (B) per‐protocol populations. Prevalence of peanut allergy in each randomized treatment group is shown by baseline eczema subgroups when data from the EAT and LEAP studies are combined in (A) ITT analysis and (B) PP analysis. Number of peanut allergy (PA) and total number of participants (*N*) are annotated at the top. Each bar is annotated with the prevalence of peanut allergy as determined by OFC at 3 years (EAT) or 5 years of age (LEAP); relative risk reduction in individual and combined studies is annotated in red. Pearson's chi‐squared was used to determine annotated *p*‐values; when expected values were less than 5, Fisher's two‐tailed exact test was used. *Fisher's *p*‐value is reported for the ITT and PP analyses in the subgroup of participants that did not have eczema at baseline as per the original statistical analysis plan. However, this analysis is expanded in Figure S4A using a model‐based causal inference approach producing a *p*‐value of .004.

The protective effect of early introduction was evident across all eczema severities with significant ITT reductions in peanut allergy prevalence in mild (85% reduction), moderate (87% reduction) and severe (67% reduction) eczema severity groups (Figure 2A). Greater reductions in peanut allergy were seen in PP analyses, with 100% reductions in children with mild and moderate eczema and a 96% reduction among the severe eczema group (Figure 2B). Among children with no eczema, there was a 36% ITT reduction (*p* = .73) and a 100% PP reduction in peanut allergy (*p* = .16) (Figure 2).

### 3.3 Peanut sensitization

In both individual and pooled study results, skin prick test sensitization to peanut was significantly reduced at 12 months among those introducing peanut early in comparison with those avoiding or introducing them after 6 months of age. IgE sensitization to peanut at 12 months of age remained similar between the randomized groups (Figure S1).

**FIGURE 3:**
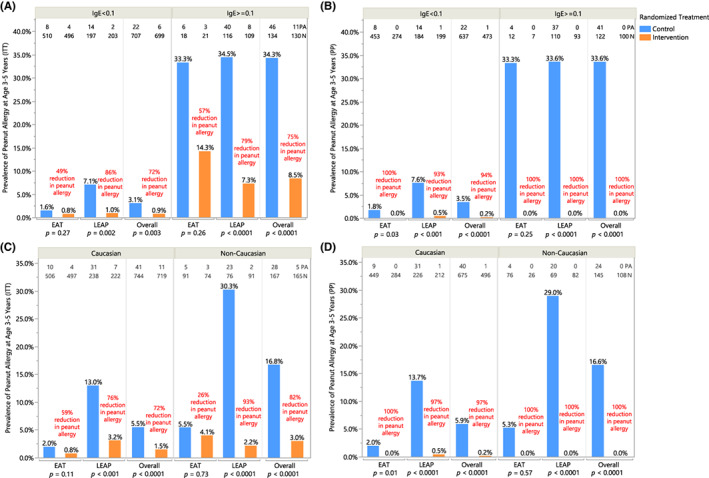
Peanut allergy prevalence by specific IgE sensitization status at enrollment—(A) intention‐to‐treat and (B) per‐protocol population; in Caucasian and non‐Caucasian children—(C) intention‐to treat and (D) per‐protocol population. Prevalence of peanut allergy in each randomized treatment group is shown in baseline sensitized (peanut‐specific IgE >= 0.1 kU/L) and unsensitized (peanut‐specific IgE <0.1 kU/L) subgroups for individual and combined studies in (A) ITT analysis and (B) PP analysis; in Caucasian and non‐Caucasian subgroups within individual and combined studies in (C) ITT analysis and (D) PP analysis. Number of peanut allergy (PA) and total number of participants (*N*) are annotated at the top. Each bar is annotated with the prevalence of peanut allergy as determined by OFC at 3 years (EAT) or 5 years of age (LEAP); relative risk reduction in individual and combined studies is annotated in red. Pearson's chi‐squared was used to determine annotated *p*‐values; when expected values were less than 5, Fisher's two‐tailed exact test was used. Fisher's *p*‐value is reported for the ITT analysis among sensitized participants in the EAT study, PP analysis in both sensitized and unsensitized subgroups in the EAT study, ITT analysis among non‐Caucasian participants in the EAT study, and PP analysis in both Caucasian and non‐Caucasian subgroups in the EAT study.

In the ITT population, children with specific IgE to peanut ≥0.1 kU/L at enrollment had a 75% reduction in peanut allergy prevalence at 3–5 years of age (*p* < .0001) (Figure 3A). There was also a statistically significant reduction in peanut allergy prevalence among children not sensitized to peanut at enrollment—72% reduction, *p* = .003 (Figure 3A). In PP analyses, this reduction was greater, with 100% reduction in peanut allergy among sensitized children and a 94% reduction in non‐sensitized children (Figure 3B).

### 3.4 Ethnicity

Non‐Caucasian groups combined (approximately 20% of all participants) had an approximately threefold higher prevalence of peanut allergy compared with Caucasians (16.8% vs 5.5%, *p* < .001). However, among non‐Caucasian ethnicities, peanut allergy rates were more similar (*p* > .05) (Figure S2). The intervention's effect size was similar in the different ethnicities, and a significant reduction in peanut allergy prevalence was seen in both Caucasian and non‐Caucasian groups (72% and 82% reduction, respectively; *p* < .0001) (Figure 3C).

### 3.5 Causal effects

**FIGURE 4:**
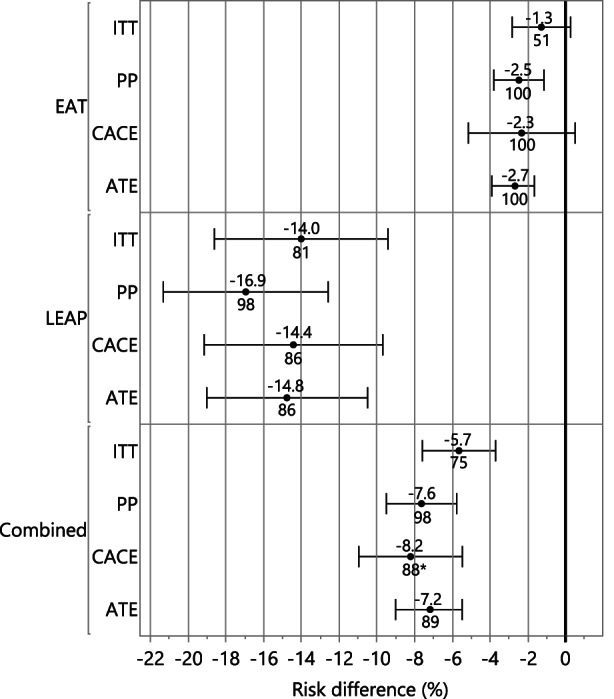
Forest plot of peanut allergy prevention effect sizes among the different study populations and analysis methods. This forest plot shows the percentage relative risk reductions in peanut allergy annotated below the marker and risk differences (x‐axis) in peanut allergy are annotated above the marker among the different cohorts (EAT, LEAP and combined studies). Relative risk reductions and risk differences are relative to the control (peanut avoidance) group and determined by intention‐to‐treat (ITT), per‐protocol (PP) and causal inference methods: complier average causal effect (CACE) and average treatment effect (ATE). Additionally, average treatment effect for the treated (ATT) and average treatment effect for the untreated (ATU) are shown in Figure S3. *Not adjusted for study; two‐staged least squares regression produces a risk difference estimate only; relative risks were computed by determining a theoretical peanut allergy rate among control participants, under the assumption of the CACE model that participants of the control group have the same probability of being non‐compliant as participants of the intervention group and being offered the intervention has no effect on the outcome. Thus, the combined relative risk under the CACE approach does not adjust for study.

Due to lower protocol compliance in the EAT study, we implemented a causal inference approach to estimate the intervention's effect on peanut allergy. In pooled analyses adjusted for study, peanut consumption demonstrated statistically significant causal effects on peanut allergy prevalence. Under all causal inference frameworks (ATE, ATT, ATU ad CACE), the predicted mean allergy incidence was lower for the group of participants that consumed peanut; ATE analysis determined an absolute risk difference in peanut allergy of −7.18% [95% CI: (−9.01, −5.49); *p* < .0001], CACE analysis: −8.22% [95% CI: (−10.96, −5.47); *p* < .0001] (Table 1 and Figure 4; ATT and ATU reported in Table S2 and Figure S3). Furthermore, consumption of peanut contributed to a relative risk reduction (RRR) of greater than 88% in all causal effect analyses of pooled data (ATE RRR of 88.8%, CACE RRR of 88.1%) (Table 1, Figure 4; ATT and ATU reported in Table S2 and Figure S3). Causal effect subgroup analyses are provided in Figures S4 and S5. Lastly, a multivariable logistic regression analysis was fit to compare the probability of peanut allergy between the EAT and LEAP cohorts after risk factor adjustment. Specifically, adjustment was made for study, randomization assignment, SCORAD group and egg allergy at baseline. Figure S6A displays the probability of peanut allergy between the EAT and LEAP cohorts according to each risk factor combination. The model‐based estimates of the risk of peanut allergy were shown to be similar between the EAT and LEAP studies, demonstrating that combining these higher and lower risk cohorts using regression adjustment adequately controlled for the heterogeneity in the outcome between these cohorts. Moreover, the analysis demonstrates significantly lower risk of peanut allergy in the consumption arm in infants without eczema and with eczema (regardless of eczema severity) as well as in the presence or absence of egg allergy (Figures S4–S6A). Figure S6B provides the raw data partitioned in a similar manner as Figure S6A to show the unmodeled peanut allergy proportions, sample sizes and number with peanut allergy in each of the risk strata, cohorts and treatment groups. Using regression adjustment, the logistic model interpolates the allergy rate across all factors in the model to give average estimates in Figure S6A, which are less influenced by the small sample sizes and sparse cases of allergy in some of the risk strata shown in Figure S6B.

**TABLE 1:** Summary of causal effect of intervention on peanut allergy outcome in EAT and LEAP and combined study‐adjusted analyses

Analysis Population	Study	*n* randomized control	*n* randomized intervention	*n* intervention received	Relative risk reduction	Risk difference % (95% CI)	*p*‐value, risk difference
ITT	EAT	597	571	315	51.2	−1.29 (0.26, −2.83)	.106
PP	EAT	525	310	315	100	−2.48 (−1.15, −3.81)	.003
CACE	EAT	597	571	315	100	−2.33 (−5.16, 0.5)	.105
ATE	EAT	597	571	315	100	−2.68 (−3.92, −1.66)	<.0001
ITT	LEAP	314	314	305	81.5	−14.01 (−9.41, −18.62)	<.0001
PP	LEAP	295	294	305	98.0	−16.95 (−12.58, −21.31)	<.0001
CACE	LEAP	314	314	305	86.2	−14.43 (−19.15, −9.69)	<.0001
ATE	LEAP	314	314	305	85.9	−14.77 (−19.01, −10.49)	<.0001
ITT	Combined	911	885	620	74.6	−5.65 (−3.71, −7.59)	<.0001
PP	Combined	820	604	620	97.9	−7.64 (−5.77, −9.5)	<.0001
CACE	Combined	911	885	620	88.1^a^	−8.22 (−10.96, −5.47)	<.0001
ATE	Combined	911	885	620	88.8	−7.18 (−9.01, −5.49)	<.0001

*Note*: Average treatment effect (ATE) was estimated through regression and propensity score adjustment methods. The ATE estimates the average treatment effect among the entire population, assuming each participant is able to receive the intervention. Complier average causal effect (CACE) analysis used an instrumental variable approach, with two‐staged least squares regression and active participation in the intervention as the predictor and randomized treatment assignment as the instrument.

^a^ Not adjusted for study. Two‐staged least squares regression produces a risk difference estimate only; relative risks were computed by determining a theoretical peanut allergy rate among control participants, under the assumption of the CACE model that participants of the control group have the same probability of being non‐compliant as participants of the intervention group and being offered the intervention has no effect on the outcome. Thus, the combined relative risk under the CACE approach does not adjust for study.

### 3.6 Age of introduction

**FIGURE 5:**
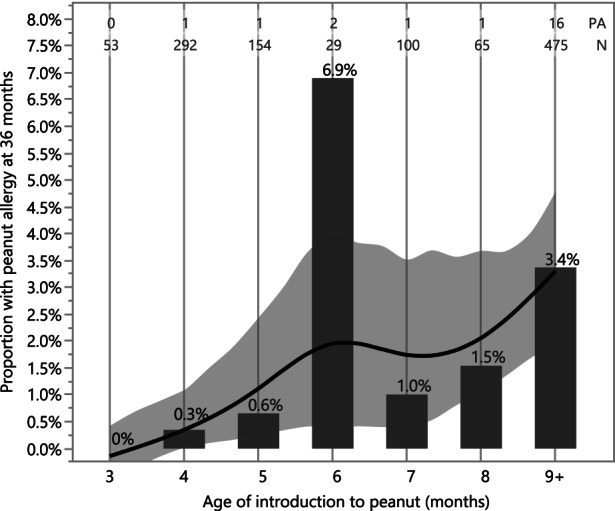
Prevalence of peanut allergy by age of introduction to peanut in the EAT study. The prevalence of peanut allergy at 36 months is shown according to the month of peanut introduction in the EAT cohort with available data (*n* = 1168) irrespective of randomization group. Those starting to consume peanut at 9 months of age or older or who never introduced peanut were grouped into a single 9+ category. The smoothed regression line and bootstrapped confidence intervals are created with a cubic spline to visualize the prevalence of peanut allergy conditional on when peanut was introduced into the diet. The adherent EAT participants randomized to the early introduction group (EIG) are shown in the 3–5 months bins whereas the non‐adherent participants are dispersed along with the standard introduction participants in the 6 to 9+ bins. The number of subjects with peanut allergy (PA), the total sample size (*N*) and the proportion (%) with peanut allergy are annotated above each bar. The large risk of peanut allergy apparent in the 6‐months category may be an artefact of the study design where participants were asked to consume before the 6‐months time point resulting in an artificially low sample size.

In an exploratory analysis, we examined the association between age of introduction of peanut and the prevalence of peanut allergy at 36 months in the EAT study (Figure 5). This analysis looked at age of introduction irrespective of the intervention group to which the subject was randomized. All but one participant introducing peanut before 6 months of age were randomized to the Early Introduction Group (EIG); however, 71 (13%) of EIG participants introduced peanut after the key early introduction period, at 6 months or later. Similarly, one Standard Introduction Group (SIG) participant introduced peanut before 6 months of age; however, there was a large amount of variability in age of introduction within the SIG, which introduced peanut, at parental discretion, at 6 months and beyond. The analysis presented in Figure 5 uses the variability in the combined randomized groups from EAT to investigate the association between age of introduction and peanut allergy. The overlaid regression line shows an increase in prevalence of peanut allergy at 3 years of age with increasing age of introduction.

## 4 DISCUSSION

In order to maximize the use of data from the EAT and LEAP studies and assess the efficacy of intervention in important subgroups, we undertook a patient‐level meta‐analysis. The EAT study included participants with varying risk, while LEAP was limited to high‐risk participants. To account for these differences, we included ethnicity, baseline eczema, egg allergy and baseline IgE in our models. A recent systematic review and meta‐analysis concluded that there was moderate evidence to support the early introduction of peanut as a method of reducing peanut allergy prevalence.^7^ However, this approach has limitations, for example a meta‐analysis cannot adjust for the varying severity profiles of the participants in the two studies. This European Union‐funded iFAAM project has successfully harmonized data from two large RCTs on early peanut introduction and analyzed pooled, individual‐level data on 1943 children from normal and high‐risk populations. While the combined population does not completely reflect the general population, the pooled analysis increases the power to analyze subgroups (e.g. eczema severity levels, sensitization groups and ethnic groups) and allows for risk factor adjustment within the integrated dataset. This approach diminishes the need for an impractically large trial in low‐risk infants.

In summary, the ITT analysis of pooled data showed a 75% reduction in peanut allergy prevalence among children randomized to consume peanut from an early age; moreover, significant reductions were demonstrated in peanut allergy across all eczema severity levels, sensitization groups and ethnic groups. Importantly, a causal inference analysis approach (ATE) demonstrated a significant benefit of the intervention among those without eczema, who make up a majority of children at risk for peanut allergy across the whole population. Moreover, a non‐randomized analysis of age of introduction in the EAT cohort demonstrates an association between earlier age of introduction and increased efficacy (Figure 5). For the first time, these results show, using integrated participant‐level data, that early peanut introduction is successful and generalizable to a wider, multi‐ethnic population and provides a strong basis for a broad public health measure. This is now supported by the recently published PreventADALL RCT in a general population in Sweden and Norway, which demonstrated an odds ratio of 0·4 (95% confidence interval 0·2 to 0·8) for peanut allergy with early introduction of peanut products.^19^

Specialist guidelines often base their recommendations purely on the ‘real world effectiveness’ that intention‐to‐treat analyses provide, whilst ignoring per‐protocol analyses. For example, while the EAT PP analysis showed 100% efficacy (*p* = .003), this analysis was not taken into account by the EAACI Guidelines committee, as their selection criteria was limited to consider only ITT analyses.^1^ We argue that the primary ITT EAT study results are ‘diluted by non‐adherence’, and the intervention's actual efficacy is evident using a PP analysis.

The reluctance to accept per‐protocol analyses as evidence for specialist guidelines has been justified by the concern of introducing bias, since randomization is not always preserved (e.g. more atopic participants could be disproportionately dropped from the intervention arm post‐randomization and thus excluded from per‐protocol analysis, resulting in bias). However, the use of newer causal inference methods have the ability to mitigate this bias with the use of propensity scores, regression adjustment and instrumental variable analysis. It is remarkable that the 2021 Nobel Prize for economics was awarded to David Card, Joshua Angrist and Guido Imbens for, ‘Answering causal questions using observational data’. In this award, Section 1.3 ‘Causal effects in a world with imperfect compliance and individual heterogeneity’, the committee comments that, ‘Imperfect compliance with treatment assignment makes it more difficult to identify the average effect of treatment, in particular when causal effects vary in the population under study … An ITT analysis thus provides an unbiased estimate of the effect of the treatment assignment in the study population, but not the actual causal effect of the treatment itself’.^20^ Using the methods pioneered by these scholars, we have attempted to address the discordance between the ITT results observed in EAT, where compliance was clearly imperfect (48%), with the results observed in LEAP, where compliance was very high (92%).

A comparison of naïve PP analyses with the newer causal inference approaches demonstrated consistency of the intervention's efficacy when adhered to. Peanut consumption reduced the risk of PA by 98% (*p* < .0001) in the pooled PP analysis. The causal inference multivariable analysis showed an 89% average treatment effect (ATE) relative risk reduction, *p* < .0001. Lower adherence (49%) in the EAT ITT population with no eczema at enrollment may have diluted the effectiveness of treatment in this underpowered, lower risk subgroup (prevalence of peanut allergy 1%).^21^, ^22^ However, a multivariable causal inference analysis approach (ATE) estimated a complete (100%) and statistically significant (*p* = .004) reduction in PA in children without eczema (Figures S4A and S5A). The consistency of the different causal effect estimates, and the similarity of these estimates to that reported for the PP analyses, provides evidence that over 85% of allergy can be prevented with early introduction of peanut.

The main strength of our study was the integration and validation of individual‐level data from two large randomized controlled trials (Figure 1), enabling us for the first time to determine efficacy of the intervention across the whole range of risk groups that comprise a normal population. Furthermore, the large number of diagnoses based on food challenge (91%) validates the estimates of allergy prevalence, and the harmonization of criteria used to diagnose food allergy and sensitization adds consistency to the data presented.

These findings are not without limitations. Firstly, the LEAP and EAT study populations were selected very differently (high and normal risk populations respectively) and followed up to aged 5 and 3 years, respectively. However, the lower risk EAT study is a heterogeneous population with both low‐ and high‐risk participants. A causal inference analysis with adjustment for risk factors such as eczema and egg allergy controls for these differences and shows the intervention to be effective. Secondly, the discrepancy between the PP and ITT results, especially among infants at lower risk of allergy, points to a difference between the intervention's ‘idealized’ efficacy (per‐protocol) and its ‘real world’ effectiveness (ITT).^23^ An intervention may be very efficacious (e.g. folate supplementation to prevent neural tube defects) but if its uptake is low, it will not be effective. While cultural and social norms are likely to play an important role in early feeding of peanuts, these preferences can change following the publication and dissemination of trial results showing efficacy of peanut introduction.^4^ Soriano (2019) showed a three‐fold increase in peanut introduction in Australia by age 1 year in 2018 compared with 2007–2011 (from 30% to 90%), which coincided with changes to national infant feeding guidelines the following publication of the LEAP study.^24^, ^25^ Good adherence to early dietary introduction of peanut products is essential for a successful peanut allergy prevention strategy.

Another weakness of our findings is that the association between age of peanut introduction and prevalence of allergy in the EAT study is, at least partially, an un‐randomized (i.e. observational) comparison. Therefore, this analysis may be confounded by other, unaccounted for factors. A similar analysis was previously performed in the LEAP population.^26^, ^27^ However, this analysis showed no association between age of introduction and prevention of allergy. This apparent discrepancy in the age‐dependent effect in EAT and the age‐independent effect in LEAP on the interventional efficacy can be explained for two reasons. Firstly, in the LEAP study the intervention was adhered to and prevented peanut allergy across the entire age range^26^, ^27^; whereas in EAT, less peanut was consumed, and protocol adherence was low (48%). Secondly, at the LEAP screening visit 76 out of the 899 participants did not meet the inclusion criteria as they were considered already peanut allergic (peanut SPT >4 mm). These excluded infants were significantly older than the rest of the LEAP screening cohort (mean age 8.3 months, SD 1.88),^12^ and it was therefore not possible to prevent peanut allergy in this older group of infants.

The results of this pooled analysis provide new evidence for the efficacy of early introduction of peanut in children with all degrees of eczema severity and moreover demonstrates efficacy in those without eczema. Moreover, this efficacy is demonstrated irrespective of ethnic group, peanut sensitization status and presence of egg allergy. These results suggest that recommending early consumption of peanut as a prevention strategy be broadly applied to the entire population, rather than targeting selected higher risk groups. Moreover, our results point to increased efficacy of the intervention with an age of introduction below 6 months, calling into question recommendations for exclusive breastfeeding during the first 6 months of life in resource‐rich regions. Utilizing individual‐level data from all RCTs of peanut introduction to date, these new analyses thus strengthen the evidence that underlie the EAACI guidelines recommending early introduction of peanut to the general population.

## AUTHOR CONTRIBUTIONS

Kirsty Logan, Henry T. Bahnson, Joanna Craven, Kirsten Beyer, Dianne E Campbell, E N Clare Mills and Gideon Lack conceptualized and designed the study. George Du Toit, Graham Roberts, Kirsty Logan, Michael R Perkin, Joanna Craven, Henry T. Bahnson, Ronald van Ree, E N Clare Mills, Johanna Bellach and Gideon Lack acquired the data. Henry T. Bahnson and Alyssa Ylescupidez analyzed the data. Kirsty Logan, Henry T. Bahnson, Alyssa Ylescupidez, Michael R Perkin, George Du Toit, Graham Roberts, Gideon Lack interpreted the data and drafted and revised the manuscript. Kirsten Beyer, Graham Roberts, Dianne E Campbell, Ronald van Ree and Gideon Lack acquired the funding.

## FUNDING INFORMATION

The main components of this analysis were funded by the European Union (Integrated Approaches to Food Allergen and Allergy Risk Management (iFAAM), Grant Agreement No: 312147). The EAT Study was jointly funded by the UK Food Standards Agency (FSA, contract code T07051) and the Medical Research Council (MRC, grant MC_G1001205). Additionally, we would like to thank the Davis Foundation. The skin‐related aspects of the EAT study were supported by the UK National Institute for Health Research (NIHR). The LEAP Study was supported by grants from the National Institute of Allergy and Infectious Diseases (NO1‐AI‐15416, UM1AI109565 and HHSN272200800029C); Food Allergy Research and Education; the Medical Research Council and Asthma UK; the United Kingdom Department of Health, through a National Institute for Health Research comprehensive Biomedical Research Centre award to Guy's and St. Thomas's NHS Foundation Trust, in partnership with King's College London and King's College Hospital NHS Foundation Trust; the National Peanut Board; and the United Kingdom Food Standards Agency.

## CONFLICT OF INTEREST

HB reports grants from the National Institute of Allergy and Infectious Diseases (NIAID, NIH) and consulting fees from DBV Technologies, outside the submitted work. KB reports institutional grants from the European Union during the conduct of the study and lecture fees from Aimmune, Allergopharma, Bencard, Danone/Nutrica, Infectopharm, Meda Pharma/Mylan, Nestle, ThermoFisher as well as consulting fees from Aimmune, ALK, Bausch & Lomb, Bencard, Danone/Nutrica, DBV, Hipp, Hycor, Infectopharm, Mabylon, Meda Pharma/Mylan, Nestle, Novartis, outside the submitted work. DEC receives a part‐time salary from the DBV Technologies as VP Clinical Development and Medical Affairs, receives institutional funding from the National Health and Medical Research Council of Australia and is on the Advisory Board of AllerGenis. The BEAT study was supported by the Ilhan Food Allergy Foundation and the Children's Hospital at Westmead Allergy and Immunology Research Fund. GdT reports grants from the National Institute of Allergy and Infectious Diseases (NIAID, NIH), Food Allergy & Research Education (FARE), MRC & Asthma UK Centre, UK Dept of Health through NIHR, Action Medical Research and National Peanut Board. Scientific Advisory Board member Aimmune, investigator on pharma‐sponsored allergy studies (Aimmune and DBV Technologies), shareholder in DBV technologies, and scientific advisor to Aimmune, DBV and Novartis. ENM reports grants from European Union, during the conduct of the study; grants from Reacta Biotech Ltd, other from Reacta Biotech Ltd, outside the submitted work. In addition, ENM has a patent Patents pending and Founder shares in Reacta Biotech Ltd. GR reports a grant from the European Union during the conduct of the study. RvR reports grants from the Dutch Science Foundation, Health Holland and the European Commission, during the conduct of the study; consultancy for HAL Allergy BV, Citeq BV and Angany Inc., speaker's fees from HAL Allergy BV and ThermoFisher Scientific, outside the submitted work. GL reports grants from the National Institute of Allergy and Infectious Diseases (NIAID, NIH), Food Allergy & Research Education (FARE), MRC & Asthma UK Centre, UK Dept of Health through NIHR, National Peanut Board (NPB), UK Food Standards Agency (FSA), Action Medical Research, the Davis Foundation during the conduct of the study; shareholder in DBV Technologies and Mighty Mission Me, scientific advisor for Novartis, Sanofi‐Genyzme, Regeneron, ALK‐Abello, outside the submitted work. No other disclosures were reported.

## Supporting information


Appendix S1

## References

[1] Halken S , Muraro A , de Silva D , et al. European academy of allergy and clinical immunology food allergy and anaphylaxis guidelines group. EAACI guideline: preventing the development of food allergy in infants and young children (2020 update). Pediatr Allergy Immunol. 2021;32(5):843‐858. 33710678 10.1111/pai.13496

[2] Committee on Toxicity of Chemicals in Food CpatE, Department of Health . COT Consumer Products and the Environment–Peanut Allergy. DoH. Crown Copyright; 1998.

[3] Kleinman RE . American Academy of Pediatrics recommendations for complementary feeding. Pediatrics. 2000;106(5):1274. 11061819

[4] du Toit G , Katz Y , Sasieni P , et al. Early consumption of peanuts in infancy is associated with a low prevalence of peanut allergy. J Allergy Clin Immunol. 2008;122(5):984‐991. 19000582 10.1016/j.jaci.2008.08.039

[5] Du Toit G , Roberts G , Sayre PH , et al. Randomized trial of peanut consumption in infants at risk for peanut allergy. N Engl J Med. 2015;372(9):803‐813. 25705822 10.1056/NEJMoa1414850 PMC4416404

[6] Perkin MR , Logan K , Tseng A , et al. Randomized trial of introduction of allergenic foods in breast‐fed infants. N Engl J Med. 2016;374(18):1733‐1743. 26943128 10.1056/NEJMoa1514210

[7] Ierodiakonou D , Garcia‐Larsen V , Logan A , et al. Timing of allergenic food introduction to the infant diet and risk of allergic or autoimmune disease. A systematic review and meta‐analysis. JAMA. 2016;316(11):1181‐1192. 27654604 10.1001/jama.2016.12623

[8] Perkin MR , Bahnson HT , Logan K , et al. Factors influencing adherence in a trial of early introduction of allergenic food. J Allergy Clin Immunol. 2019;144(6):1595‐1605. 31812183 10.1016/j.jaci.2019.06.046 PMC6904906

[9] Togias A , Cooper S , Acebal M , et al. Addendum guidelines for the prevention of peanut allergy in the United States: report of the National Institute of Allergy and Infectious Diseases–sponsored expert panel. J Allergy Clin Immunol. 2017;139(1):29‐44. 28065278 10.1016/j.jaci.2016.10.010 PMC5226648

[10] Murray EJ , Caniglia EC , Swanson SA , Hernández‐Díaz S , Hernán MA . Patients and investigators prefer measures of absolute risk in subgroups for pragmatic randomized trials. J Clin Epidemiol. 2018;103:10‐21. doi:10.1016/j.jclinepi.2018.06.009 29966732 PMC6175611

[11] Hernán MA , Robins JM . Per‐protocol analyses of pragmatic trials. N Engl J Med. 2017;77(14):1391‐1398. 10.1056/NEJMsm1605385 28976864

[12] Du Toit G , Roberts G , Sayre P , et al. Identifying infants at high risk of peanut allergy: the learning early about peanut allergy (LEAP) screening study. J Allergy Clin Immunol. 2013;131(1):135‐143. 23174658 10.1016/j.jaci.2012.09.015

[13] Perkin MR , Logan K , Marrs T , et al. Enquiring about tolerance (EAT) study: feasibility of an early allergenic food introduction regimen. J Allergy Clin Immunol. 2016;137(5):1477‐1486. 26896232 10.1016/j.jaci.2015.12.1322 PMC4852987

[14] Bang H , Robins JM . Doubly robust estimation in missing data and causal inference models. Biometrics. 2005;61:962‐973. 16401269 10.1111/j.1541-0420.2005.00377.x

[15] Lunceford JK , Davidian M . Stratification and weighting via the propensity score in estimation of causal treatment effects: a comparative study. Stat Med. 2004;23:2937‐2960. 15351954 10.1002/sim.1903

[16] Wooldridge JM . Econometric Analysis of Cross Section and Panel Data. 2nd ed. MIT Press; 2004.

[17] SAS Institute Inc . SAS/STAT 14.2 User's Guide. SAS Institute Inc.; 2016.

[18] Yang Jiang and Dylan Small . (2014). ivpack: Instrumental Variable Estimation. R package version 1. 2. https://CRAN.R‐project.org/package=ivpack

[19] Skjerven HO , Lie A , Vettukattil R , et al. Early food intervention and skin emollients to prevent food allergy in young children (PreventADALL): a factorial, multicentre, cluster‐randomised trial. Lancet. 2022;399(10344):2398‐2411. 35753340 10.1016/S0140-6736(22)00687-0

[20] The Royal Swedish Academy of Sciences . Scientific Background on the Sveriges Riksbank Prize in Economic Sciences in Memory of Alfred Nobel. 2021. https://www.nobelprize.org/uploads/2021/10/advanced‐economicsciencesprize2021.pdf

[21] Iglesia EGA , Kim EH . Low‐risk infants may still benefit from allergenic food consumption. J Allergy Clin Immunol. 2020;145(4):1305. 10.1016/j.jaci.2020.01.016 32111421

[22] Perkin MR , Bahnson HT , Lack G . Letter of response to Iglesia et al. “Low‐risk infants may still benefit from allergenic food consumption”. J Allergy Clin Immunol. 2020;145(4):1305‐1306. 10.1016/j.jaci.2020.01.016 32111421

[23] Haynes B . Can it work? Does it work? Is it worth it? BMJ. 1999;319:652‐653. 10480802 10.1136/bmj.319.7211.652 PMC1116525

[24] Soriano VX , Peters RL , Ponsonby AL . Earlier ingestion of peanut after changes to infant feeding guidelines: the EarlyNuts study. J Allergy Clin Immunol. 2019;144(5):1327‐1335. 31401287 10.1016/j.jaci.2019.07.032

[25] Joshi PA , Smith J , Vale S , Campbell DE . The Australasian Society of Clinical Immunology and Allergy infant feeding for allergy prevention guidelines. Med J Aust. 2019;210(2):89‐93. 30636277 10.5694/mja2.12102

[26] Greenhawt MJ , Fleischer DM , Atkins D , Chan ES . The complexities of early Peanut introduction for the practicing allergist. J Allergy Clin Immunol Pract. 2016;4(2):221‐225. 26968960 10.1016/j.jaip.2015.12.016

[27] Lawson K , Bahnson HT , Brittain E , et al. Letter of response to Greenhawt et al. 'LEAPing through the looking glass: secondary analysis of the effect of skin test size and age of introduction on peanut tolerance after early peanut introduction'. Allergy. 2017;72(8):1267‐1271. 28691223 10.1111/all.13127 PMC5796413

