# Supplementary material for: Early introduction of peanut reduces peanut allergy across risk groups in pooled and causal inference analyses
Source: Allergy. 2022 Dec 11;78(5):1307–18. doi: 10.1111/all.15597 (PMC10202125; doi:10.1111/all.15597)
Supplement: Supplementary file 1 — Appendix S1 [file ALL-78-1307-s001.docx]

**Supplementary Appendix**

**Early introduction of peanut reduces peanut allergy across risk groups in pooled and causal inference analyses**

Kirsty Logan*^a^, Henry T. Bahnson*^b^, Alyssa Ylescupidez^b^, Kirsten Beyer^c^, Johanna Bellach^c^, Dianne E Campbell^d^, Joanna Craven^a^, George Du Toit^a^, E N Mills^e^, Michael R Perkin^f^, Graham Roberts^g^, Ronald van Ree^h^, and Gideon Lack^a^

**km**

From ^a^the Department of Women and Children's Health, School of Life Course and Population Sciences, King’s College London; ^b^ Immune Tolerance Network, Benaroya Research Institute, Seattle, *United States of America*; ^c^Charité Universitätsmedizin Berlin, Berlin, Germany;  ^d^ Children's Hospital at Westmead, Sydney, Australia; ^e^the School of Biological Sciences, Division of Infection, Immunity and Respiratory Medicine, Manchester Academic Health Science Centre, Manchester Institute of Biotechnology, University of Manchester; ^f^the Population Health Research Institute, St George's, University of London; ^g^University of Southampton and Southampton NIHR Biomedical Research Centre, Southampton, United Kingdom;  ^h^the Departments of Experimental Immunology and of Otorhinolaryngology, Amsterdam University Medical Centers, location AMC, Amsterdam.

*These authors contributed equally to this manuscript

**Table S1 Baseline demographics and study-specific cohort size.**

|  | **Control** (%)  (n=972) | **Intervention** (%)  (n=971) | **Total**  (n=1,943) |
| --- | --- | --- | --- |
|  |  |  |  |
| **Study: EAT** | 651 (67) | 652 (67) | 1303 (67) |
| **LEAP** | 321 (33) | 319 (33) | 640 (33) |
|  |  |  |  |
| **Sex: male** | 547 (56) | 490 (50) | 1037 (53) |
| **female** | 425 (44) | 481 (50) | 906 (47) |
|  |  |  |  |
| **Ethnicity: Caucasian** | 791 (81) | 783 (81) | 1574 (81) |
| **Black** | 45 (5) | 44 (4) | 89 (5) |
| **Mixed/other** | 136 (14) | 143 (15) | 279 (14) |
|  |  |  |  |
| **Mean SCORAD at baseline** | 13.4 (SD 19.6) | 13.1 (SD 19.0) | 13.3 (SD 19.3) |
|  |  |  |  |
| **Eczema severity at baseline:** |  |  |  |
| **No eczema** | 503 (52) | 499 (51) | 1002 (52) |
| **Mild (SCORAD 1-14)** | 160 (16) | 172 (18) | 332 (17) |
| **Moderate (SCORAD 15-39)** | 181 (19) | 176 (18) | 357 (18) |
| **Severe (SCORAD≥40)** | 127 (13) | 124 (13) | 251 (13) |
|  |  |  |  |
| **IgE sensitization to peanut at baseline:**  **<0.1 kU/L**  **>0.1 kU/L** | 759 (85)  137 (15) | 773 (85)  134 (15) | 1532 (85)  271 (15) |
| **Peanut Allergy at 3-5 years of age:** | 69 (7.6) | 17(1.9) | 86 (4.8) |
|  |  |  |  |

**Table S2 Summary of causal effect of intervention on peanut allergy outcome in EAT and LEAP and combined study-adjusted analyses.** ^‡^**Not adjusted for study.**

| Analysis Population | Study | n randomized control | n randomized intervention | n intervention received | Relative risk reduction | Risk difference  % (95% CI) | p-value, risk difference |
| --- | --- | --- | --- | --- | --- | --- | --- |
| ITT | EAT | 597 | 571 | 315 | 51.2 | -1.29  (0.26, -2.83) | 0.106 |
| PP | EAT | 525 | 310 | 315 | 100 | -2.48  (-1.15, -3.81) | 0.003 |
| CACE | EAT | 597 | 571 | 315 | 100 | -2.33  (-5.16, 0.5) | 0.105 |
| ATE | EAT | 597 | 571 | 315 | 100 | -2.68  (-3.92, -1.66) | <.0001 |
| ATT | EAT | 597 | 571 | 315 | 100 | -2.47  (-3.67, -1.26) | <.0001 |
| ATU | EAT | 597 | 571 | 315 | 100 | -2.77  (-3.93, -1.6) | <.0001 |
| ITT | LEAP | 314 | 314 | 305 | 81.5 | -14.01  (-9.41, -18.62) | <.0001 |
| PP | LEAP | 295 | 294 | 305 | 98.0 | -16.95  (-12.58, -21.31) | <.0001 |
| CACE | LEAP | 314 | 314 | 305 | 86.2 | -14.43  (-19.15, -9.69) | <.0001 |
| ATE | LEAP | 314 | 314 | 305 | 85.9 | -14.77  (-19.01, -10.49) | <.0001 |
| ATT | LEAP | 314 | 314 | 305 | 86.4 | -14.62  (-18.95, -10.29) | <.0001 |
| ATU | LEAP | 314 | 314 | 305 | 85.5 | -14.91  (-19.36, -10.45) | <.0001 |
| ITT | Combined | 911 | 885 | 620 | 74.6 | -5.65  (-3.71, -7.59) | <.0001 |
| PP | Combined | 820 | 604 | 620 | 97.9 | -7.64  (-5.77, -9.5) | <.0001 |
| CACE | Combined | 911 | 885 | 620 | 88.1^‡^ | -8.22  (-10.96, -5.47) | <.0001 |
| ATE | Combined | 911 | 885 | 620 | 88.8 | -7.18  (-9.01, -5.49) | <.0001 |
| ATT | Combined | 911 | 885 | 620 | 87.9 | -8.62  (-10.97, -6.27) | <.0001 |
| ATU | Combined | 911 | 885 | 620 | 89.4 | -6.38  (-7.97, -4.78) | <.0001 |

Average treatment effect (ATE), average treatment effect for the treated (ATT), and average treatment effect for the untreated (ATU), were estimated through regression and propensity score adjustment methods. The ATE estimates the average treatment effect among the entire population, assuming each participant is able to receive the intervention. The ATT estimates the treatment effect among the distribution of participants who received the treatment (‘per-protocol adherent’). Conversely, the ATU estimates the treatment effect among the distribution of subjects who did not receive the treatment (non-per- protocol adherent). The estimates were adjusted for ethnicity, baseline eczema severity, and baseline peanut sensitisation as covariates in each study separately and in the pooled study-adjusted analysis. Complier average causal effect (CACE) analysis used an instrumental variable approach, with two-staged least squares regression and active participation in the intervention as the predictor and randomized treatment assignment as the instrument.

^‡^Two-staged least squares regression produces a risk difference estimate only; relative risks were computed by determining a theoretical peanut allergy rate among control participants, under the assumption of the CACE model that participants of the control group have the same probability of being non-compliant as participants of the intervention group and being offered the intervention has no effect on the outcome. Thus, the combined relative risk under the CACE approach does not adjust for study.

**Figure S1 Peanut SPT and sIgE sensitization at 12 months of age in the per-protocol population.**

**
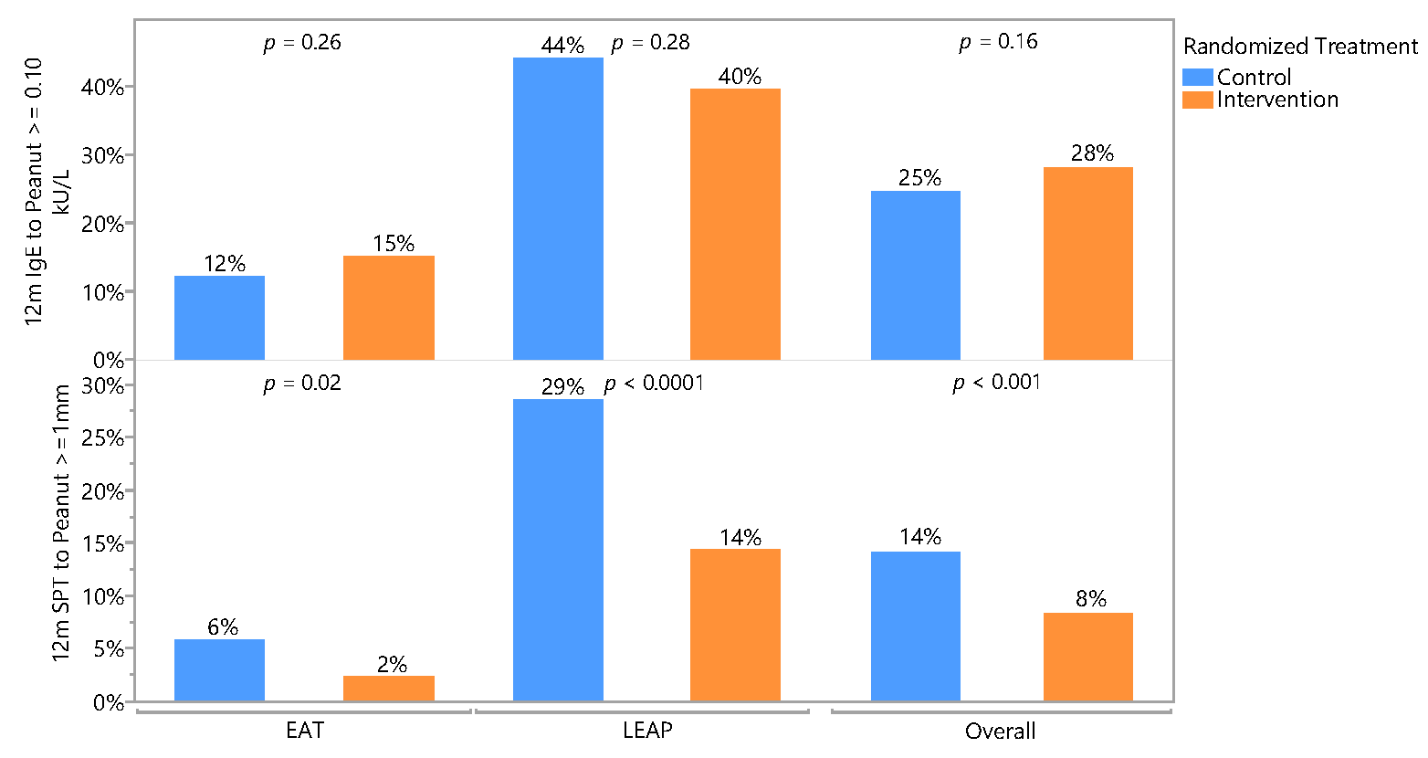
**

**Figure S2 Peanut allergy prevalence in ethnicity groups – a) Intention-to-treat and b) Per-protocol population.
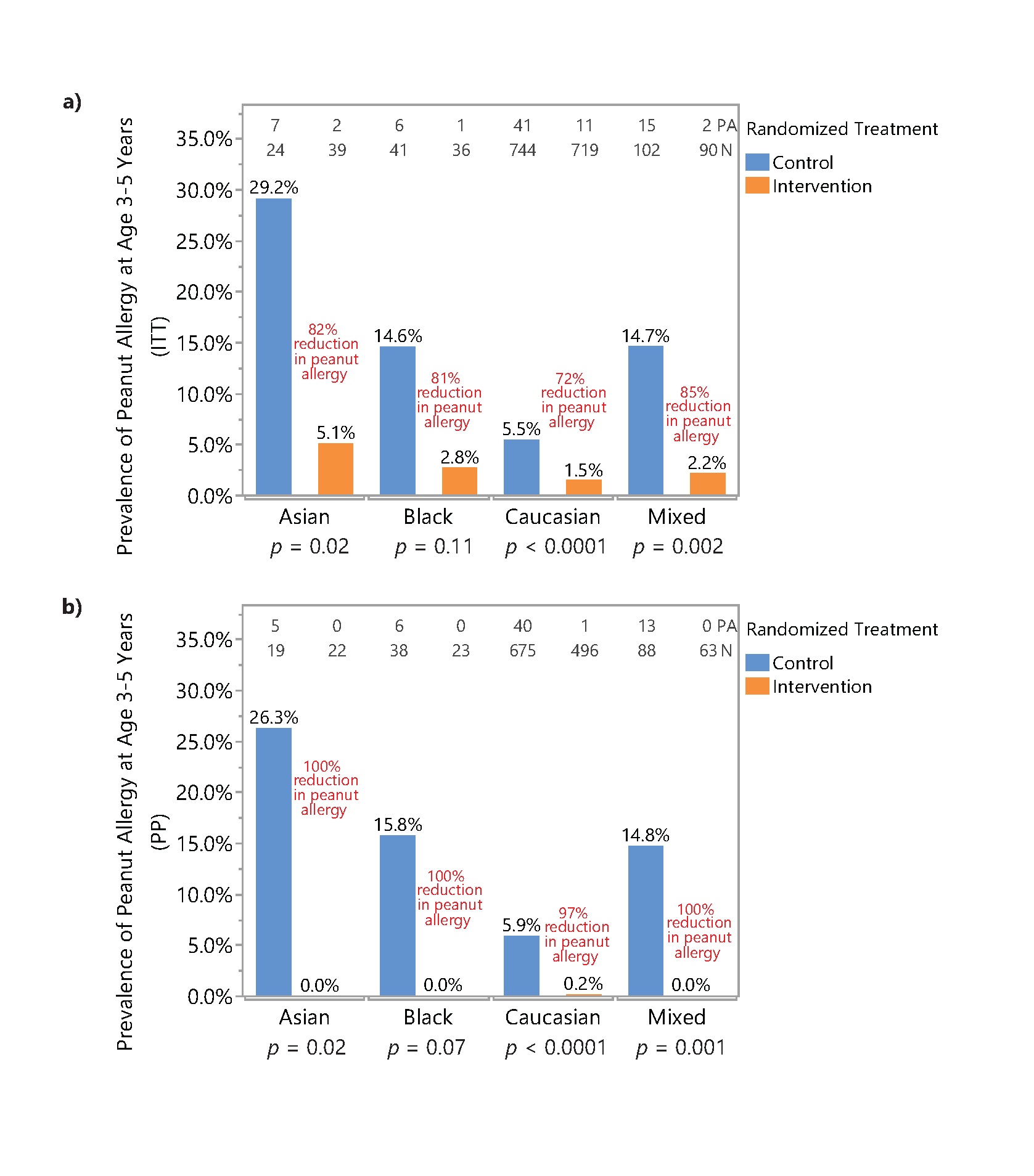
**

**Figure S****3 Forest plot of peanut allergy prevention effect sizes among the different study populations and analysis methods; *not adjusted for study.**

**
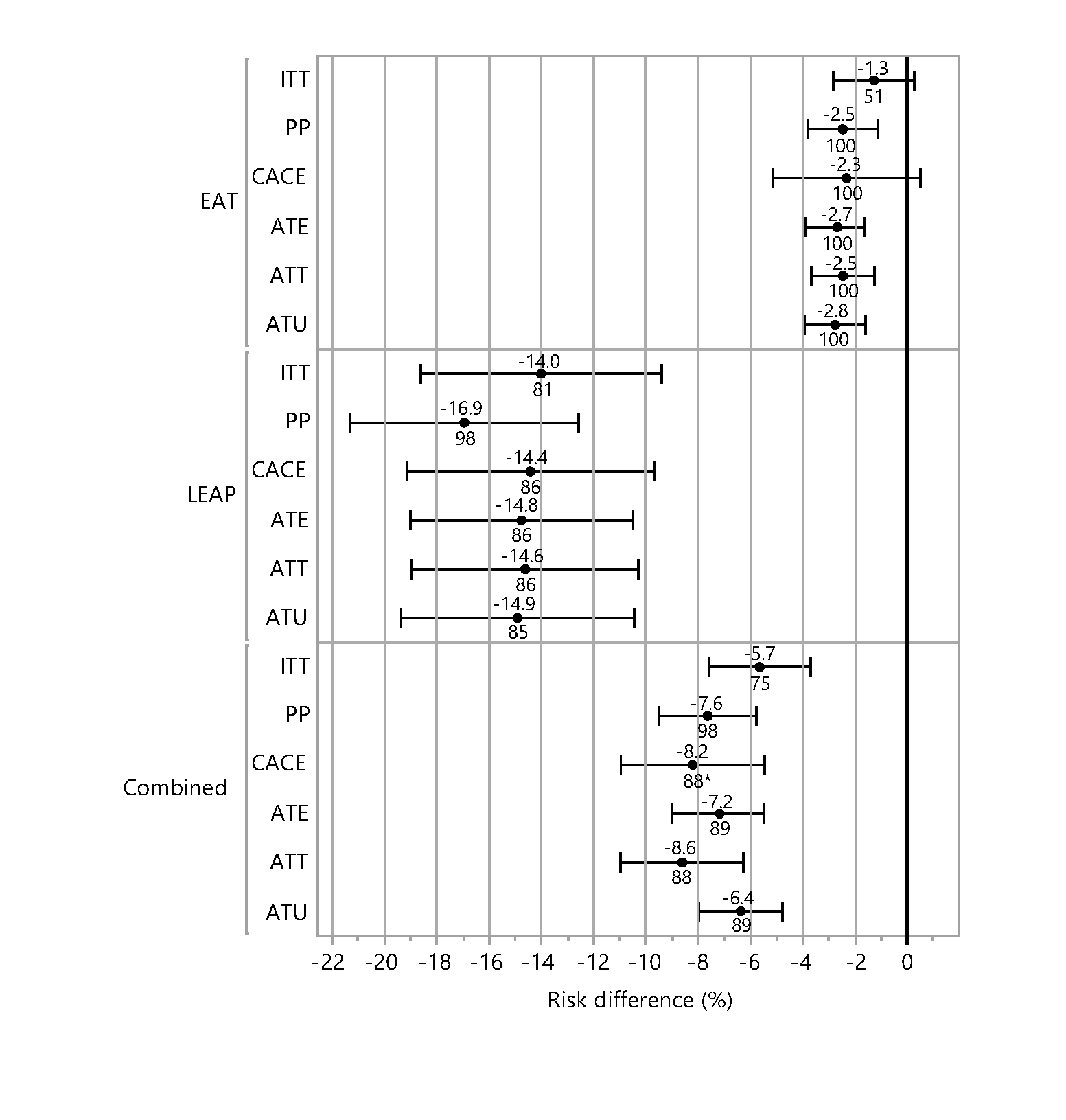
**

**Figure S4 Estimated prevalence of peanut allergy (determined by average treatment effect approach) in subgroups by a) eczema severity at baseline, b) baseline IgE sensitization to peanut, and c) ethnicity.**

**
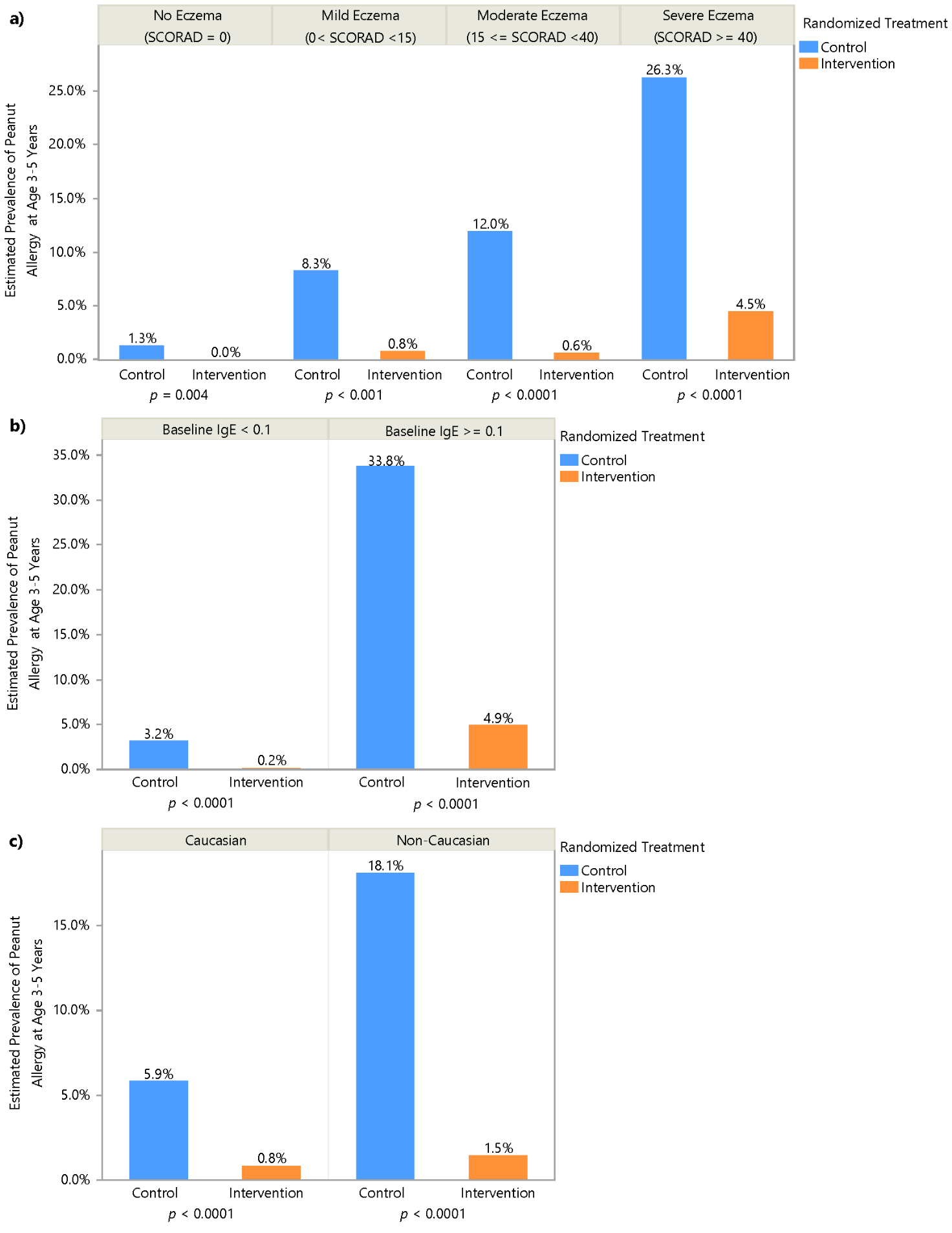
**

**Figure S5 Causal effects of early introduction of peanut (absolute risk difference and 95% CI), as determined by ATE and CACE causal framework, in subgroups by a) eczema severity at baseline, b) baseline IgE sensitization to peanut, and c) ethnicity.**

**
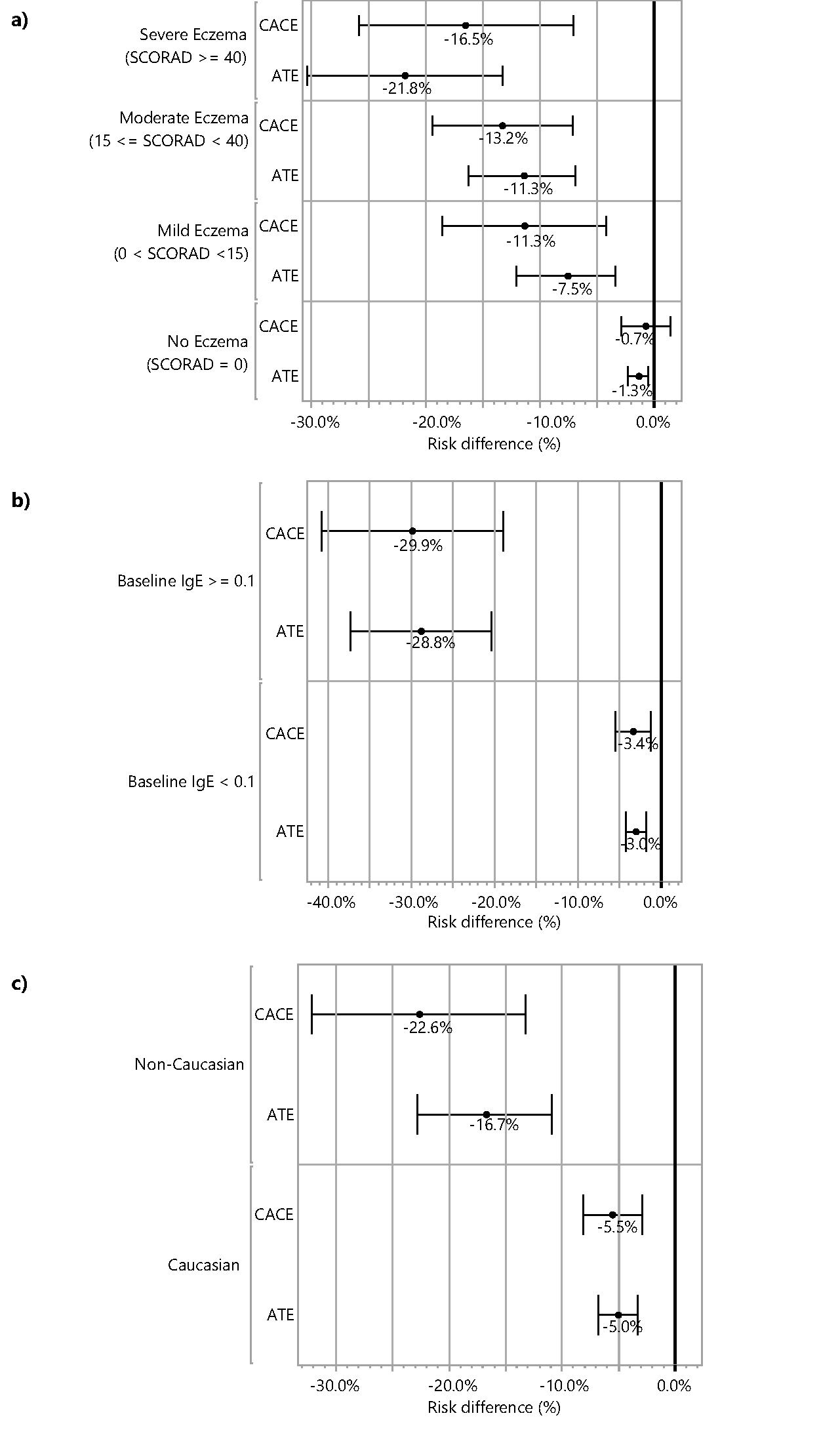
**

**Figure S6 (a) Probability of peanut allergy after risk factor adjustment between the EAT and LEAP cohorts (b) unadjusted proportion with peanut allergy stratified by the combination of risk factors, cohorts, and randomization assignment.**

**a)**

1. The probability of peanut allergy is shown on the y-axis according to the combinations of risk factors (X-axis and row partition) and randomization assignment (column partition). Probability estimates were produced using a multivariable logistic regression model adjusting for study (p=0.863), randomization assignment (p<.0001), SCORAD group (p<.0001), and egg allergy at baseline (p<.0001). The similar estimates of peanut allergy risk between the EAT and LEAP studies across the predictor combinations demonstrates the appropriateness of combining these cohorts using regression adjustment.
2. The raw unadjusted proportions with peanut allergy are shown similarly to panel a. The annotated “sum” row on the top gives the sum total of the number with peanut allergy in each bar. The annotated “N” row on the bottom gives the sample size in each bar. Dividing the sum by N gives the corresponding proportion with allergy.
